# Untargeted Metabolomic Analysis of the Effects and Mechanism of Nuciferine Treatment on Rats With Nonalcoholic Fatty Liver Disease

**DOI:** 10.3389/fphar.2020.00858

**Published:** 2020-06-09

**Authors:** Huantian Cui, Yuting Li, Min Cao, Jiabao Liao, Xiangguo Liu, Jing Miao, Hui Fu, Ruiwen Song, Weibo Wen, Zhaiyi Zhang, Hongwu Wang

**Affiliations:** ^1^Shandong Provincial Key Laboratory of Animal Cell and Developmental Biology, School of Life Sciences, Shandong University, Qingdao, China; ^2^College of Traditional Chinese Medicine, Tianjin University of Traditional Chinese Medicine, Tianjin, China; ^3^College of Integrated Chinese and Western Medicine, Tianjin University of Traditional Chinese Medicine, Tianjin, China; ^4^Department of Emergency, Jiaxing Hospital of Traditional Chinese Medicine, Jiaxing, China; ^5^Department of Integrated Traditional and Western Medicine, Tianjin Second People’s Hospital, Tianjin, China; ^6^College of Management, Tianjin University of Traditional Chinese Medicine, Tianjin, China; ^7^Yunnan Provincial Hospital of Traditional Chinese Medicine, Kunming, China

**Keywords:** nonalcoholic fatty liver disease, nuciferine, metabolic disorder, metabolomic analysis

## Abstract

Metabolomic analysis has been used to characterize the effects and mechanisms of drugs for nonalcoholic fatty liver disease (NAFLD) at the metabolic level. Nuciferine is an active component derived from *folium nelumbinis* and has been demonstrated to have beneficial effects on a high-fat diet (HFD) induced hepatic steatosis model. However, the effect of the altered metabolites of nuciferine on NAFLD has not yet been elucidated. In this study, we established a NAFLD rat model using HFD and treated with nuciferine. The lipid content levels, pro-inflammatory cytokines, and oxidative stress were investigated to access the therapeutic effects of nuciferine. Additionally, the metabolic regulatory mechanisms of nuciferine on NAFLD were analyzed using untargeted metabolomics. Gene expression of the key enzymes related to the changed metabolic pathways following nuciferine intervention was also investigated. The results showed that nuciferine treatment significantly reduced the body weight, levels of lipids, and liver enzymes in the blood and improved the hepatic steatosis in the NAFLD rat model. Nuciferine treatment also increased the activities of superoxide dismutase (SOD) and glutathione peroxidase (GSH-Px) and decreased the levels of methane dicarboxylic aldehyde (MDA) in the liver. Nuciferine treatment decreased the serum levels of interleukin (IL)-6, IL-1β, and tumor necrosis factor-alpha (TNF-α) and upregulated the gene expression of *IL-6*, *IL-1β*, and *TNF-α* in the liver. Metabolomic analysis indicated a metabolism disorder in the NAFLD rat model reflected in a dysfunction of the glycerophospholipid, linoleic acid, alpha-linolenic acid, arginine and proline metabolism. Conversely, treatment with nuciferine improved the metabolic disorder in the NAFLD rat model. Nuciferine treatment also regulated the gene expression of key enzymes related to the glycerophospholipid, linoleic acid, and alpha-linolenic acid metabolism pathways in the liver. In conclusion, our study demonstrated an amelioration of the metabolic disorders following nuciferine treatment in NAFLD rat model. Our study contributes to the understanding of the effects and mechanisms of drugs for complex diseases using metabolomic analysis and experimental approaches.

## Introduction

Nonalcoholic fatty liver disease (NAFLD) is a chronic metabolic disease that is characterized by steatosis of the hepatocytes and dyslipidemia. Prolonged NAFLD could cause chronic metabolic diseases, including diabetes, liver ﬁbrosis, liver cirrhosis, and hepatocellular carcinoma (HCC) (Wilkins et al., 2013; Masarone et al., 2014). Currently, the prevalence of NAFLD is becoming a global health concern. Epidemiology studies showed that the morbidity of NAFLD is 20–30% worldwide and could reach up to 75% in the obese population (Younossi et al., 2019).

Metabolomics can assist with screening the modifications to the endogenous metabolites in the disease models in order to understand the pathogenesis of diseases at the metabolic level. The dysfunction of the metabolic processes in NAFLD has been reviewed previously (Yu et al., 2017). In addition, metabolomics could provide a systematic view of the metabolites in the presence or absence of treatment and further clarify the potential mechanisms associated with this treatment. Quercetin could ameliorate NAFLD in rats *via* regulating the alpha-linolenic acid, linoleic acid, arachidonic acid (AA), and bile acid metabolism pathways (Xu et al., 2019). Silybin could modulate the lipid, polyol, and amino acid metabolisms, as well as the urea and the tricarboxylic acid cycles in a NAFLD mouse model (Sun et al., 2020).

Nuciferine, an active component derived from *folium nelumbinis*, has been demonstrated to ameliorate dyslipidemia and regulate the expression of genes involved in lipogenesis, free fatty acid (FFA) β-oxidation, FFA infiltration, inflammation and oxidative stress on a high-fat diet (HFD)-induced hepatic steatosis model (Guo et al., 2013). In addition, studies have shown the beneficial effects of nuciferine on diabetic rats by regulating lipid metabolism (Ning et al., 2019). *In vitro* studies also revealed that nuciferine inhibited lipid accumulation and downregulated the expression of Per-Arnt-Sim Kinase (PASK) in oleic acid-induced hepatic steatosis in HepG_2_ cells (Zhang et al., 2015). However, the altered metabolites of nuciferine on NAFLD were poorly studied.

In this study, we established a NAFLD rat (*Rattus norvegicus*) model using HFD and treated with nuciferine. The therapeutic effects of nuciferine on NAFLD were evaluated by investigating the levels of lipid contents, pro-inflammatory cytokines, and oxidative stress. Additionally, untargeted metabolomic was used to analyze the metabolic regulatory mechanisms of nuciferine on NAFLD.

## Materials and Methods

### Reagents

Nuciferine (C_19_H_21_NO_2_; molecular weight, 295.38 Da; purity ≥ 98%) was purchased from Sichuan Weikeqi Biological Technology Co., Ltd. (Sichuan, China). HFD (17.7% sucrose, 17.7% fructose, 19.4% protein, and 40% fat) was obtained from Beijing Huafukang Bioscience Co., Ltd. (Beijing, China). Triglyceride (TG), total cholesterol (TC), high-density lipoprotein cholesterol (HDL-C), low-density lipoprotein cholesterol (LDL-C), aspartate aminotransferase (AST), alanine aminotransferase (ALT), superoxide dismutase (SOD), methane dicarboxylic aldehyde (MDA), and glutathione peroxidase (GSH-Px) assay kits test kits were obtained from Nanjing Jiancheng Biological Engineering Institute (Nanjing, China). Oil Red O Staining Kit was purchased from Solarbio Biotechnology Co., Ltd. (Beijing, China). Total RNA extraction, first-strand cDNA reverse transcription, polymerase chain reaction (PCR) kits and primers were obtained from TianGen Biotechnology Co., Ltd. (Beijing, China). Rat interleukin (IL)-6, IL-1β, and tumor necrosis factor-alpha (TNF-α) enzyme-linked immunosorbent assay (ELISA) kits were obtained from Shanghai BlueGene Biotech Co., Ltd. (Shanghai, China).

### Induction of NAFLD Rat Model

The NAFLD model was established as previously described (Li et al., 2020). Briefly, rats received HFD containing 26% carbohydrate (sucrose as carbohydrate source), 26% protein, and 35% fat for 12 weeks. Lard, rich in saturated fatty acids, was used as the source of fat in the HFD.

### Animals and Treatment

Male Sprague-Dawley (SD) rats, 6–8 weeks old and 190–210 g weight, were purchased from Huafukang Animal Co., Ltd. (Beijing, China). They were housed in a controlled environment (12-h light/dark cycle, 21 ± 2°C with a relative humidity of 45 ± 10%) with *ad libitum* access to food and water. All animal experiments were approved by the Animal Ethics Committee at Tianjin University of Traditional Chinese Medicine.

**Figure 1:**
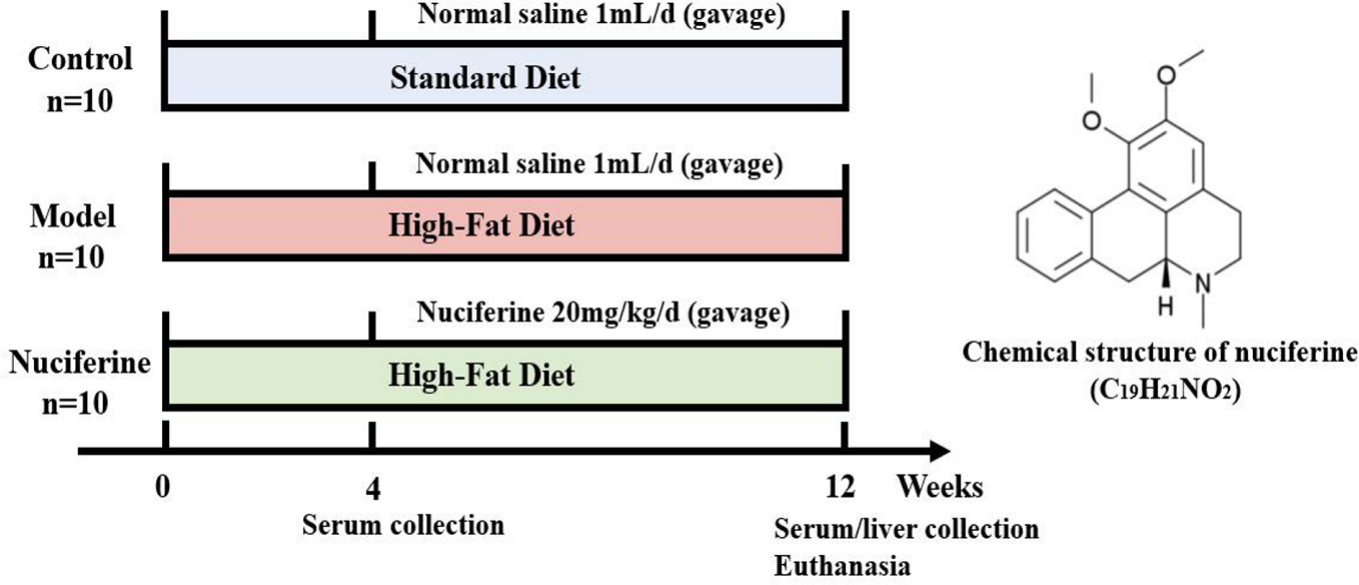
Overview of the experimental design for all groups.

After the acclimatization for 3 days, all animals were randomly divided into 3 groups (n = 10): control, experimental model, and nuciferine-treated groups. Rats in the control group were fed with standard laboratory chow containing 59.4% carbohydrate (cereal grain as the main carbohydrate source), 20% protein, and 4.8% fat and the other groups were given HFD to generate a NAFLD model. After 4 weeks of HFD treatment, rats in the nuciferine-treated group received a gavage of nuciferine (20 mg/kg body weight) once per day for 8 weeks (Wang et al., 2016; Ye et al., 2018), meanwhile, the same volume of saline solution was given intragastrically in the control and experimental model groups once per day for 8 weeks. The liver index was investigated at the end of 8 weeks after nuciferine treatment. The liver index was calculated using the following formula: liver index (%) = liver weight (g)/body weight (g) × 100 (**Figure 1**).

### Serum Biochemical Markers Assay

Serum samples were collected at both week 4 and week 12 after HFD treatment for the biochemical assays. Briefly, rats were anaesthetized with 10% chloral hydrate solution (0.3 ml/100g body weight, intraperitoneally) and blood was harvested by a capillary glass tube from the inner canthus. The serum was obtained by centrifuging at 3,000 rpm for 15 min. The levels of serum ALT, AST, TG, TC, HDL-C, and LDL-C were assayed according to the manufacturer’s instructions provided by Nanjing Jiancheng Biological Engineering Institute (Nanjing, China) and the absorbance value was detected using a microplate reader (Varioskan Flash, Thermo Fisher, Massachusetts, USA).

### H&E Staining

At the end of 12 weeks following nuciferine treatment, the rats were euthanized and the rat livers were removed immediately and fixed in 10% formalin, dehydrated, and embedded in paraffin wax. The tissues were cut into 5 µm sections using a microtome (RM2125, Leica, Buffalo Grove, USA). Sections were subsequently stained with hematoxylin and eosin (H&E) as previously described (Cui et al., 2017). The extent of steatosis, lobular inflammation and hepatocyte ballooning was determined using the NAFLD activity score (NAS) (Kobyliak and Abenavoli, 2014). Scores ranged from 0 to 8 (total score), representing the sum of the scores for the severity of steatosis, lobular inflammation, and hepatocyte ballooning (**Table 1**).

**Table 1:** NAFLD Activity Score (NAS) (Kobyliak and Abenavoli, 2014).

Feature Score	Score	Description
Steatosis	0	0–5% hepatocytes involved
	1	5–33% of hepatocytes involved
	2	33–66% hepatocytes involved
	3	>66% hepatocytes involved
Hepatocyte ballooning	0	none
	1	Few; inapparent
	2	Easily noted; many
Lobular inflammation	0	None
	1	<2 foci per × 200 field
	2	2–4 foci per × 200 field
	3	>4 foci per × 200 field

The extent of NAFLD was evaluated by NAS. The score ranged from 0 to 8 (total score), representing the sum of scores of steatosis, hepatocyte ballooning, and lobular inflammation.

### Oil Red O Staining

Livers were sectioned using a sliding vibratome (CM3050S, Leica, Buffalo Grove, USA) to obtain the coronal cryostat sections (20 μm thick). The sections were then stained with Oil Red O following the manufacturer’s instructions. The staining extent of Oil Red O was analyzed and quantified using Image J. The integrated optical density (IOD) was then observed. The mean optical density (MOD) was calculated using the following formula: MOD = IOD/sum area.

### Liver Biochemical Analysis

The liver tissues (0.1 g) were weighed and immersed in 900 µl normal saline followed by ultrasonic trituration and centrifugation at 3,000 rpm for 15 min to obtain liver tissue homogenates. The obtained supernatants were used to measure the SOD and GSH-Px activities and the MDA level according to the manufacturer’s instructions provided by Nanjing Jiancheng Biological Engineering Institute (Nanjing, China).

### Cytokine Quantiﬁcation by Enzyme-Linked Immunosorbent Assay (ELISA)

Following 12 weeks of nuciferine treatment, the levels of IL-6, IL-1β, and TNF-α in the serum were measured using ELISA according to the manufacturer’s instructions (Shanghai BlueGene Biotech Co., Ltd. China).

### RNA Isolation and Real-Time Reverse Transcription Quantitative Polymerase Chain Reaction (qPCR)

Total RNA was isolated from the rat livers using an RNA extraction kit (TianGen Biotechnology Co., Ltd. Beijing, China). The first-strand cDNA was synthesized using 1 µg of total RNA according to the manufacturer’s instructions (TianGen Biotechnology Co., Ltd. Beijing, China). Real-time reverse transcription quantitative polymerase chain reaction (qPCR) was used to detect the expression of *IL-6*, *IL-1β*, *TNF-α*, *phosphatidylethanolamine N-methyltransferase (PEMT)*, *lecithin cholesterol acyltransferase (LCAT)*, *phosphatidylserine synthase (PTDSS) 1*, *PTDSS2*, *phospholipase A2 (PLA2)*, *fatty acid desaturase 2 (FADS2)*, *CYP (cytochrome P-450) 2C*, *CYP2E1*, *CYP3A4*, and *arginase 2 (ARG2)* in the liver as previously described (Cui et al., 2018). All samples were performed in triplicate and detected using a BioRad iQ5 Detection System. *β-actin* was used as a loading control. Quantiﬁcation was performed using the 2^−△△CT^ method (Livak and Schmittgen, 2002). The sequences of the primers were listed in **Table 2**.

**Table 2:** Primer sequences of target genes for rats.

Genes	Primer sequence (5′-3′)
*β-actin*	Forward: TCTTCCAGCCTTCCTTCCTG
	Reverse: CACACAGAGTACTTGCGCTC
*IL-6*	Forward: CTCATTCTGTCTCGAGCCCA
	Reverse: TGAAGTAGGGAAGGCAGTGG
*IL-1β*	Forward: GGGATGATGACGACCTGCTA
	Reverse: TGTCGTTGCTTGTCTCTCCT
*TNF-α*	Forward: GAGCACGGAAAGCATGATCC
	Reverse: TAGACAGAAGAGCGTGGTGG
*PEMT*	Forward: ATGTGGTAGCAAGGTGGGAG
	Reverse: AGGCCCAGGAAGTAGATGGT
*LCAT*	Forward: ACTACCAGAAGCTGGCAGGA
	Reverse: AGCCATCAATGAAGTGGTCC
*PLA2*	Forward: AAACAAGGCAGGCCCTTGAAC
	Reverse: TGATCACAACTGCTAGCAACAGGAG
*PTDSS1*	Forward: CGGGAAGATCAAGAGAGCTG
	Reverse: ATGGATGACTGGCTTGGAAC
*PTDSS2*	Forward: TTCCAACCTACAAGGGCAAG
	Reverse: GAATGTGTTCAGCTCTGCCA
*FADS2*	Forward: ATCTGCCCTACAACCACCAG
	Reverse: AGTTGAGGAAAACCAGGGCT
*CYP2C*	Forward: AACAGGCATCGAGCATCTCT
	Reverse: AGCAAGAGCAAAAGCCCATA
*CYP3A4*	Forward: TTCGATGTGGAGTGCCATAA
	Reverse: CTTTCCCCATAATCCCCACT
*CYP2E1*	Forward: TGAAAAAGCCAAGGAACACC
	Reverse: TCTCAGAGTTGTGCTGGTGG
*ARG2*	Forward: TCGTGTATCCTCGCTCAGTG
	Reverse: CATGAGCATCAACCCAGATG

### Metabolomics Study

The changes observed in the metabolites in serum following nuciferine treatment for 12 weeks were screened using liquid chromatography–mass spectrometry (LC-MS) (King et al., 2019). The detailed protocols used for sample preparation, LC-MS and data analysis are described below.

### Sample Preparation

Ten μl of 2-chloro-l-phenylalanine (0.3 mg/ml) and Lyso PC17:0 (0.01 mg/ml) were dissolved in methanol and added to 100 μl of the serum samples. Subsequently, 300 μl of the protein precipitation solution containing methanol and acetonitrile (2/1, v/v) was added to each sample, followed by vortexing for 1 min. The mixture was then ultrasonicated in ice-cold water for 10 min and was incubated at −20°C for 30 min. Following incubation, the mixture was centrifuged at 13,000 rpm for 10 min and 300 μl of supernatant was collected, dried, and redissolved in 400 μl of a methanol/water solution (1/4, v/v). Following redissolving, the samples were centrifuged at 13,000 rpm for 10 min and 150 μl of supernatant was collected and filtered using a 0.22-μm filter and stored at −80°C before LC-MS analysis. Samples used for quality control (QC) were prepared by mixing aliquots of all samples in the same volume.

### Liquid Chromatography–Mass Spectrometry

Metabolic profiling using electrospray ionization (ESI)-positive and ESI-negative ion modes for each sample was analyzed using the ACQUITY UHPLC system (Waters Corporation, Milford, USA) and the AB SCIEX Triple TOF 5600 system (AB SCIEX, Framingham, MA). An ACQUITY UPLC BEH C18 column (100 mm × 2.1 mm, 1.7 um) was used for the analytes with both positive and negative modes. The column temperature was maintained at 45°C. The chromatographic analysis of each sample was conducted with the mobile phase, including (A) water (containing 0.1% formic acid, v/v) and (B) methanol (containing 0.1% formic acid, v/v). The flow rate of the mobile phase was 0.25 ml/min and the injection volume was 2 μl. Separation was conducted as follows: 0 min, 2% B; 2 min, 2% B; 7 min, 98% B; 14 min, 98% B; 14.1 min, 2% B; and 16 min, 2% B.

Data acquisition was performed for both positive and negative ionization scan modes (m/z ranges from 100 to 1,200) *via* the ESI source. The detailed parameters of mass spectrometry (MS) for the positive ionization mode were as follows: spray voltage of 3,500 V, probe heater temperature at 300°C (+), sheath gas flow rate of 30 arbitrary units, auxiliary gas flow rate of 10 arbitrary units and capillary temperature at 320°C. The detailed parameters of MS for the negative ionization mode were as follows: spray voltage of 3,500 V, probe heater temperature at 350°C, sheath gas flow rate of 30 arbitrary units, auxiliary gas flow rate of 10 arbitrary units, and capillary temperature at 320°C. The QCs were injected every 10 samples throughout the analytical run to ensure the data accuracy.

### Data Processing and Analysis

The acquired LC-MS raw data were analyzed using the Progenesis QI software (Nonlinear Dynamics, Newcastle, UK) to obtain three- dimensional data, including m/z, peak retention time and peak intensities. The primary parameters were set as follows: precursor tolerance, 5 ppm; product tolerance, 10 ppm; and product ion threshold, 5%. The metabolites were identified and detected using the Human Metabolome Database (HMDB)^a^, Lipidmaps (v2.3),^b^ and METLIN^c^ databases. Subsequently, the peaks with a missing value (ion intensity = 0) in more than 50% of samples were filtered out. The metabolites were scored based on the three-dimensional data and metabolites with a compound score less than 30 were screened out.

The metabolic alterations occurring between the control and the experimental model group as well as between the experimental model group and the nuciferine-treated group were visualized using the principle component analysis (PCA) and the orthogonal partial least squares discriminant analysis (OPLS-DA) models (Kind et al., 2009). Seven-round cross-validation and 200 times modeling for the response permutation testing (RPT) were used to guard against over-fitting in the analysis models based on the R^2^ and Q^2^ values. Variable influence on projection (VIP) values for each variable were obtained from the OPLS-DA model. The normalized peak areas for each metabolite were represented as mean ± standard deviation (mean ± SD) and were analyzed *via* a two-tailed Student’s *t*-test. Metabolites with a VIP > 1 and *p* < 0.05 among each group were considered to be differential metabolites. Differential metabolites in the control and the experimental model groups with a fold change (FC) > 1.25 or FC < 0.80 were selected for the metabolic pathway analysis. The metabolic pathways for the differential metabolites in the control and model groups were analyzed using the MetaboAnalyst software^d^. The pathway library for the Kyoto Encyclopedia of Genes and Genomes (KEGG)^e^ was selected (Sun et al., 2020).

a: http://www.hmdb.ca/
b: http://www.lipidmaps.org/
c: http://metlin.scripps.edu/
d: https://www.metaboanalyst.ca/
e: https://www.kegg.jp/

### Statistics

All data are reported as the mean ± SD for the independent experiments. Statistical diﬀerences between the experimental groups were examined using the analysis of variance (ANOVA) and SPSS software, version 20.0. A *p*-value < 0.05 was considered statistically signiﬁcant. Curve ﬁtting was performed using the GraphPad Prism 5 software.

## Results

### Effects of Nuciferine on HFD-Induced NAFLD Model Rats

Following 4 weeks of HFD treatment, the body weight of the rats was significantly increased in the experimental model and the nuciferine groups as compared to the control group (*p* < 0.05, respectively, **Table 3**). Furthermore, the serum levels of TG, TC, LDL-C, AST, and ALT (*p* < 0.01, respectively, **Tables 4** and **5**) were increased and the level of HDL-C (*p* < 0.05, **Table 4**) in the serum was decreased in the experimental model and nuciferine groups as compared to that of the control group.

**Table 3:** The effects of nuciferine treatment on body weight and liver index.

Group	Body weight (g)	Liver index (%)
**4 weeks after HFD treatment**		
Control	292.0 ± 22.3	–
Model	321.9 ± 27.3^#^	–
Nuciferine	324.2 ± 30.4^#^	–
**12 weeks after HFD treatment**		
Control	425.0 ± 37.7	2.82 ± 0.25
Model	461.6 ± 35.0^#^	3.28 ± 0.37^#^
Nuciferine	419.5 ± 43.8^*^	2.91 ± 0.29^*^

Control, experimental model, and nuciferine-treated (n = 10 per group) groups. Data are presented as the mean ± SD. ^#^p < 0.05 as compared to the control group; *p < 0.05 as compared to the experimental model group.

**Table 4:** Changes of blood lipid levels in NAFLD model rats.

Group	TG (mmol/L)	TC (mmol/L)	LDL-C(mmol/L)	HDL-C(mmol/L)
**4 weeks after HFD treatment**				
Control	1.94 ± 0.28	4.40 ± 0.87	0.83 ± 0.10	1.93 ± 0.35
Model	5.42 ± 0.65^##^	6.03 ± 0.59^##^	1.13 ± 0.30^##^	1.65 ± 0.21^#^
Nuciferine	5.37 ± 0.91^##^	5.91 ± 0.94^##^	1.16 ± 0.24^##^	1.61 ± 0.26^#^
**12 weeks after HFD treatment**				
Control	1.92 ± 0.30	4.46 ± 1.26	0.85 ± 0.09	2.02 ± 0.56
Model	8.74 ± 2.04^##^	7.60 ± 1.03^##^	1.41 ± 0.25^##^	1.49 ± 0.15^#^
Nuciferine	4.22 ± 1.51^**^	5.66 ± 1.51^*^	0.93 ± 0.36^*^	1.84 ± 0.37

Control, experimental model, and nuciferine-treated (n = 10 per group) groups. Data are presented as the mean ± SD. ^#^p < 0.05 as compared to the control group; ^##^p < 0.01 as compared to the control group; *p < 0.05 as compared to the experimental model group; **p < 0.01 as compared to the experimental model group.

**Table 5:** Changes in blood ALT and AST levels in NAFLD model rats.

Group	ALT (U/L)	AST (U/L)
**4 weeks after HFD treatment**		
Control	50.82 ± 7.24	70.97 ± 6.39
Model	85.23 ± 11.10^##^	120.56 ± 11.14^##^
Nuciferine	82.43 ± 8.83^##^	116.15 ± 12.67^##^
**12 weeks after HFD treatment**		
Control	48.89 ± 6.12	67.44 ± 7.46
Model	123.18 ± 11.31^##^	190.92 ± 17.26^##^
Nuciferine	65.74 ± 6.54^**^	90.46 ± 9.36^**^

Control, experimental model, and nuciferine-treated (n = 10 per group) groups. Data are presented as the mean ± SD. ^##^p < 0.01 as compared to the control group; **p < 0.01 as compared to the experimental model group.

After 8 weeks of nuciferine treatment, the body weight (*p* < 0.05) and liver index (*p* < 0.05) were increased in the experimental model group as compared to the control group. Differently, nuciferine treatment significantly decreased the body weight (*p* < 0.05) and liver index (*p* < 0.05, **Table 3**) in the NAFLD rat model. In addition, the serum levels of TG, TC, LDL-C, AST, and ALT (*p* < 0.01, respectively, **Tables 4** and **5**) were increased and the level of HDL-C (*p* < 0.05, **Table 4**) in the serum was decreased in the experimental model group as compared to that of the control group. Nuciferine treatment significantly decreased the serum levels of TG, TC, LDL-C, AST, and ALT (*p* < 0.01, *p* < 0.05, *p* < 0.05, *p* < 0.01, and *p* < 0.01, respectively, **Tables 4** and **5**) as compared to the experimental model group.

**Figure 2:**
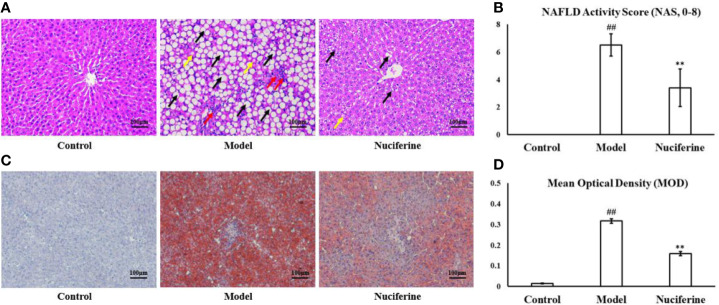
Nuciferine treatment improved the hepatic steatosis. **(A, B)** H&E staining revealed enlarged hepatocytes in the experimental model group and the nuciferine-treated rats showed only moderate steatosis and ballooning in the liver as well as a lower NAS. Black arrows indicate the steatosis of hepatocytes, red arrows indicate lobular inflammation, and yellow arrows indicate hepatocyte ballooning (200× magnification). **(C, D)** Oil Red O staining indicated that the lipid-loaded hepatocytes and the MOD of staining extents were decreased in the nuciferine-treated rats as compared to the experimental model group (200× magnification). Control, experimental model, and nuciferine-treated (n = 10 per group) groups. ^##^*p* < 0.01 as compared to the control group; **p < 0.01 as compared to the experimental model group.

H&E staining revealed enlarged hepatocytes with severe microvesicular steatosis and ballooning as well as lobular inflammation in the experimental model group, which resulted in a higher NAS compared with the control group (*p* < 0.01, **Figures 2A, B**). Furthermore, Oil Red O staining confirmed hepatosteatosis and increased lipid deposition in the experimental model group (*p* < 0.01, **Figures 2C, D**). Liver sections in the nuciferine-treated rats showed only moderate steatosis, ballooning, lower NAS (*p* < 0.01, **Figures 2A, B**) and less lipid-loaded hepatocytes (*p* < 0.01, **Figures 2C, D**) as compared to the experimental model group.

### Anti-Oxidative and Anti-Inflammatory Effects of Nuciferine on HFD-Induced NAFLD Rat Model

Previous studies have shown that oxidative stress plays an important role in the progression of NAFLD (Braud et al., 2017). Thus, we further investigated whether nuciferine inhibited oxidative stress in the NAFLD rats. The results showed that the activities of SOD and GSH-Px were lower and the level of MDA was higher in the experimental model group as compared to the control group (*p* < 0.01, respectively, **Table 6**), whereas the activities of SOD (*p* < 0.05) and GSH-Px (*p* < 0.05) were increased, while the level of MDA (*p* < 0.01) was decreased in the nuciferine-treated group as compared to the experimental model group (**Table 6**).

**Table 6:** The activities of SOD and GSH-Px and the levels of MDA in rat liver homogenate after nuciferine treatment.

Group	SOD (U/mgprot)	MDA (nmol/mgprot)	GSH-Px (U/mgprot)
Control	184.18 ± 16.49	2.43 ± 0.43	69.21 ± 1.95
Model	134.45 ± 29.44^##^	16.55 ± 1.66^##^	53.55 ± 5.80^##^
Nuciferine	161.07 ± 16.30^*^	7.57 ± 2.41^**^	60.41 ± 8.28^*^

Control, experimental model, and nuciferine-treated (n = 10 per group) groups. Data are presented as the mean ± SD. ^##^p < 0.01 as compared to the control group; *, p < 0.05 as compared to the experimental model group; **, p < 0.01 as compared to the experimental model group.

**Figure 3:**
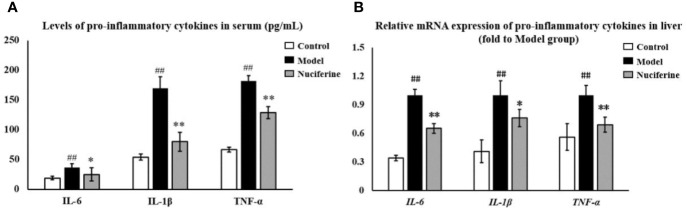
Anti-inflammatory effects of nuciferine in the NAFLD rat model. **(A)** Nuciferine treatment decreased the serum levels of IL-6, IL-1β, and TNF-α. **(B)** Nuciferine treatment downregulated the gene expression of *IL-6*, *IL-1β*, and *TNF-α* in the liver (fold to experimental model group). Control, experimental model, and the nuciferine-treated (n = 6 per group) groups. Data are presented as mean ± SD. ^##^*p* < 0.01 as compared to the control group; *p < 0.05 as compared to the experimental model group; **p < 0.01 as compared to the experimental model group.

The levels of IL-6, IL-1β, and TNF-α in the serum were investigated using ELISA. The levels of IL-6, IL-1β, and TNF-α were higher in the experimental model group compared to that of the control group (*p* < 0.01, respectively, **Figure 3A**). Nuciferine treatment decreased the levels of IL-6, IL-1β, and TNF-α in the serum as compared to the experimental model group (*p* < 0.05, *p* < 0.01, and *p* < 0.01, respectively, **Figure 3A**). We also investigated the relative mRNA expression of *IL-6*, *IL-1β*, and *TNF-α* in the liver using qPCR. The expression of *IL-6*, *IL-1β*, and *TNF-α* was higher in the experimental model group as compared to the control group (*p* < 0.01, respectively, **Figure 3B**), whereas nuciferine treatment downregulated the expression of *IL-6*, *IL-1β*, and *TNF-α* as compared to the experimental model group (*p* < 0.01, *p* < 0.05, and *p* < 0.01, respectively, **Figure 3B**).

### Multivariate Analysis of Serum Metabolomics

**Figure 4:**
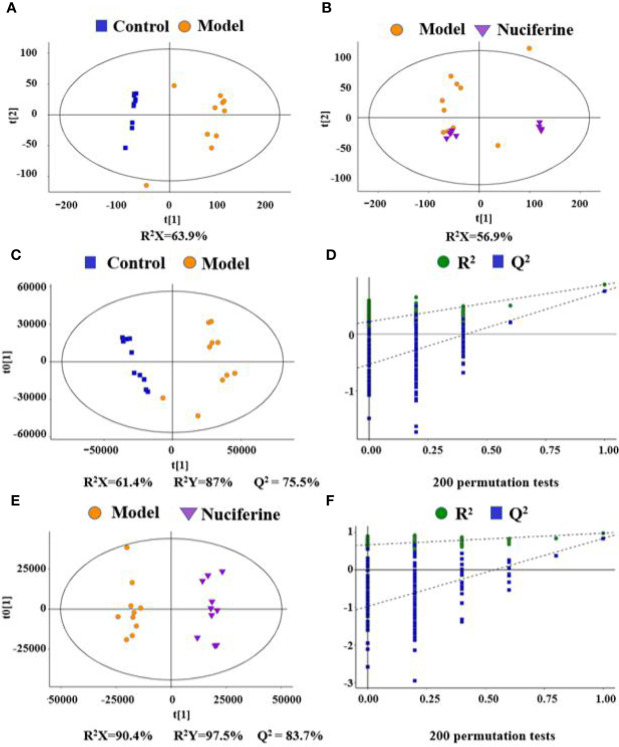
Variable analysis of the metabolomics data in each group. **(A, B)** Score plots of PCA between the control group and the experimental model group and the experimental model group and the nuciferine-treated group; **(C, D)** Score plots of OPLS-DA between the control group and the experimental model group and the corresponding coefficient of loading plots; **(E, F)** Score plots of OPLS-DA between the experimental model group and the nuciferine-treated group and the corresponding coefficient of loading plots. Control, experimental model, and nuciferine-treated (n = 10 per group) groups.

The typical based peak intensity (BPI) chromatograms of the serum samples were analyzed in positive and negative modes, respectively (**SFigures 1** and **2**). Endogenous markers in all groups were optimally separated after 16 min. PCA showed a clear group separation between the control and the experimental model groups, while an unclear distinction was obtained between the experimental model and the nuciferine-treated groups, according to the PCA model. Therefore, an OPLS-DA was performed to analyze the metabolomics data. The OPLS-DA models indicated significant metabolic variations between the control group and the experimental model group as well as between the experimental model group and the nuciferine-treated group (**Figure 4**). Likewise, seven-round cross-validation and 200 times of RPT showed that the OPLS-DA models were robust. When comparing the control group with the experimental model group, the R^2^ and Q^2^ values in the OPLS-DA model were 0.219 and −0.532, respectively. However, when comparing the experimental model group with the nuciferine-treated group, the R^2^ and Q^2^ values in the OPLS-DA model were 0.668 and −0.973, respectively (**Figure 4**).

### Identification of Differential Metabolites

**Figure 5:**
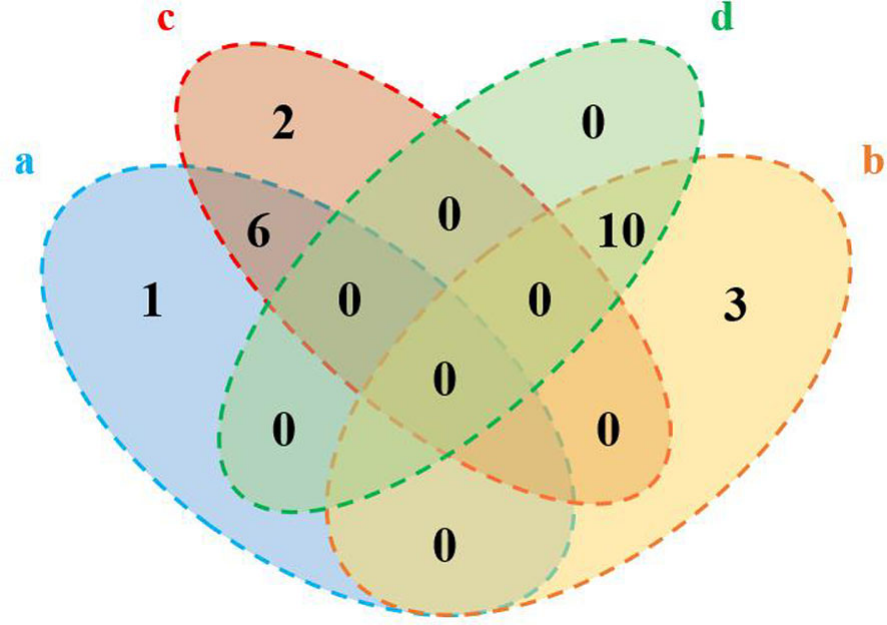
Numbers of the differential metabolites between the control group and the experimental model group and the experimental model group and the nuciferine-treated group (Venn plot). **(A)** Decreased levels in the experimental model group as compared to the control group. **(B)** Elevated levels in the experimental model group as compared to the control group. **(C)**, Elevated levels in the nuciferine-treated group as compared to the experimental model group. **(D)** Decreased levels in the nuciferine-treated group as compared to the experimental model group.

Generally, 20 differential metabolites between the control group and the experimental model group and 18 differential metabolites between the experimental model group and the nuciferine-treated group were identified (VIP > 1 and *p* < 0.05, **Figure 5**). Differential metabolites between the experimental model and the control groups were selected for the metabolic pathway analysis. Among the 20 differential metabolites between the experimental model and the control groups, phosphatidylcholine (PC), dihomo-gamma-linolenate, rumenic acid, linoleic acid, 1-acylglycerophosphocholine, alpha-linolenic acid, and eicosapentaenoic acid (EPA) were decreased. Furthermore, 12(R)-HETE, L-leucine, L-valine, L-isoleucine, phosphatidylethanolamine (PE), glycerylphosphorylethanolamine, L-proline, L-arginine, L-tryptophan, 2-hydroxycinnamic acid, trans-cinnamic acid, nicotyrine, and THTC were increased (**Table 7**).

**Table 7:** The differential serum metabolites between the experimental groups.

No.	Rt (min)	m/z	Formula	Metabolites	VIP		FC		Trend		Pathway
					M vs. C	N vs. M	M vs. C	N vs. M	M vs. C	N vs. M	
1	0.83	940.8140	C_56_H_112_NO_8_P	Phosphatidylcholine	2.44	1.87	0.62	1.29	↓^# #^	↑*	c
2	11.32	305.2484	C_20_H_34_O_2_	Dihomo-gamma-linolenate	1.08	1.44	0.52	1.49	↓^# #^	↑**	a
3	0.71	281.2460	C_18_H_32_O_2_	Linoleic acid	1.35	1.60	0.65	1.26	↓^# #^	↑*	a
4	10.99	279.2327	C_18_H_32_O_2_	Rumenic acid	4.89	6.01	0.33	2.03	↓^# #^	↑**	a
5	10.50	277.2171	C_18_H_30_O_2_	Alpha-linolenic acid	1.33	1.69	0.44	1.39	↓^#^	↑*	b
6	10.41	325.2901	C_20_H_25_D_5_O_2_	EPA (d5)	3.59	2.86	0.85	1.05	↓^# #^	↑	–
7	9.43	319.2273	C_20_H_32_O_3_	12(R)-HETE	3.84	3.99	2.06	0.40	↑^#^	↓*	–
8	2.92	132.1014	C_6_H_13_NO_2_	L-leucine	4.14	3.32	3.84	0.49	↑^# #^	↓*	–
9	1.43	118.0860	C_5_H_11_NO_2_	L-valine	2.97	2.37	3.15	0.50	↑^# #^	↓*	–
10	2.69	132.1014	C_6_H_13_NO_2_	L-isoleucine	3.15	2.48	2.48	0.51	↑^# #^	↓*	–
11	10.58	766.4845	C_45_H_70_NO_8_P	Phosphatidylethanolamine	1.41	1.24	1.39	0.86	↑^# #^	↓	c
12	10.49	570.3527	C_30_H_52_NO_7_P	1-acylglycerophosphocholine	1.26	1.08	0.34	2.24	↓^# #^	↑**	c
13	6.60	216.0622	C_5_H_14_NO_6_P	Glycerylphosphorylethanolamine	1.20	1.88	3.21	0.73	↑^#^	↓	c
14	1.06	116.0703	C_5_H_9_NO_2_	L-proline	2.51	2.01	4.37	0.52	↑^# #^	↓*	d
15	0.88	175.1181	C_6_H_14_N_4_O_2_	L-arginine	2.87	2.31	3.70	0.80	↑^# #^	↓	d
16	5.77	203.0816	C_11_H_12_N_2_O_2_	L-tryptophan	1.23	1.06	2.72	0.50	↑^# #^	↓*	–
17	2.85	182.0804	C_9_H_8_O_3_	2-hydroxycinnamic acid	4.17	3.63	3.14	0.48	↑^# #^	↓*	–
18	5.17	166.0855	C_9_H_8_O_2_	Trans-cinnamic acid	5.17	4.35	2.63	0.48	↑^# #^	↓*	–
19	5.75	159.0909	C_10_H_10_N_2_	Nicotyrine	1.17	1.00	2.40	0.46	↑^# #^	↓*	–
20	1.72	150.0576	C_5_H_8_O_2_S	THTC	2.78	2.53	3.07	0.42	↑^# #^	↓**	–
21	10.85	730.2405	C_26_H_45_NO_21_	Lacto-N-tetraose	0.95	2.37	0.87	1.20	↓^#^	↑**	–
22	10.90	339.2871	C_19_H_40_O_3_	1,2,4-Nonadecanetriol	0.96	3.02	0.95	1.23	↓	↑**	–

^#^p < 0.05 as compared to the control group; ^##^p < 0.01 as compared to the control group; *p < 0.05 as compared to the experimental model group; **p < 0.01 as compared to the experimental model group; ↑, content increased; ↓, content decreased; vs., versus; C, control group; M, experimental model group; N, nuciferine-treated group.

(a) Linoleic acid metabolism, (b) alpha-linolenic acid metabolism, (c) glycerophospholipid metabolism, and (d) arginine and proline metabolism.

### Pathway Analysis of Differential Metabolites

**Figure 6:**
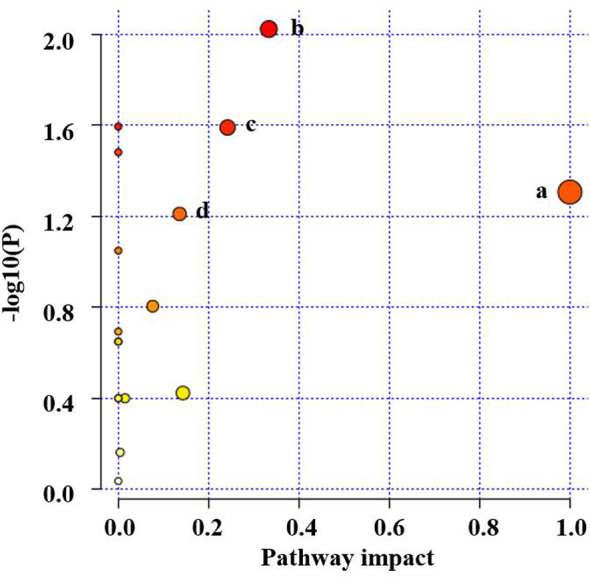
Pathway analysis for serum samples in the HFD-induced NAFLD rat model. **(A)** linoleic acid metabolism, **(B)** alpha-linolenic acid metabolism, **(C)** glycerophospholipid metabolism, and **(D)** arginine and proline metabolism.

**Figure 7:**
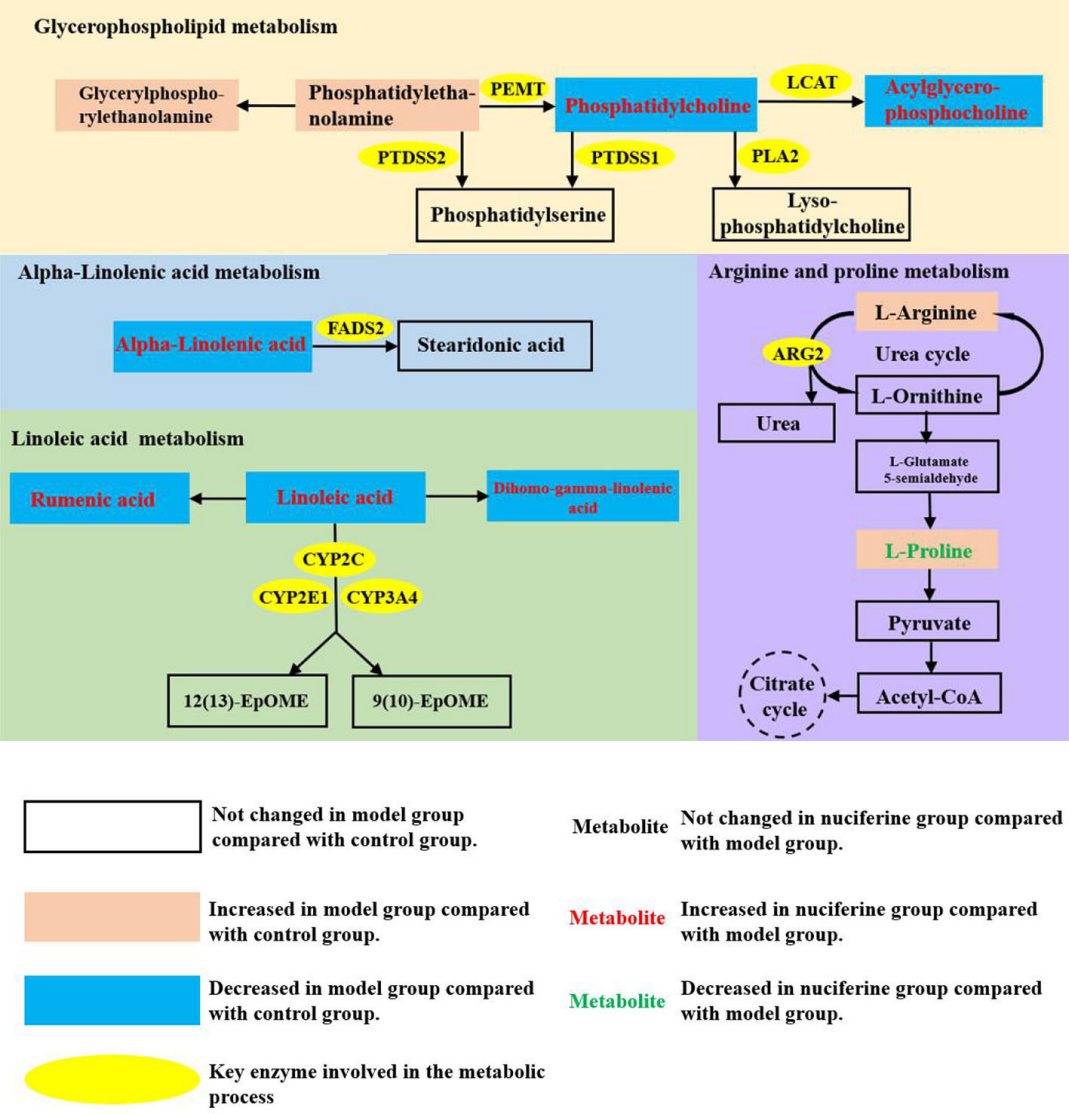
Summary schematic of significant pathways in rats with NAFLD after nuciferine treatment. 12(13)-EpOME, (12R,13S)-(9Z)-12,13-epoxyoctadecenoic acid; 9(10)-EpOME, (9R,10S)-(12Z)-9,10-epoxyoctadecenoic acid; PEMT, phosphatidylethanolamine N-methyltransferase; LCAT, lecithin cholesterol acyltransferase; PLA2, phospholipase A2; PTDSS1, phosphatidylserine synthase 1; PTDSS2, phosphatidylserine synthase 2; FADS2, fatty acid desaturase 2; CYP2C, cytochrome P-450 2C; CYP2E1, cytochrome P-450 2E1; CYP3A4, cytochrome P-450 3A4; ARG2, arginase 2.

**Figure 8:**
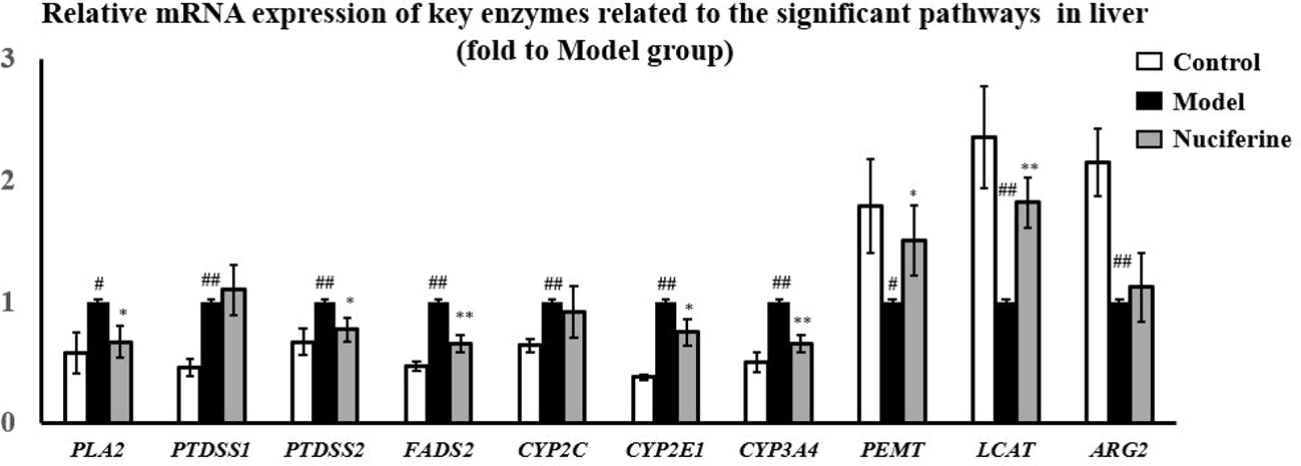
Gene expression of *PLA2*, *PTDSS2*, *FADS2*, *CYP2E1*, and *CYP3A4* was downregulated, while *PEMT* and *LCAT* was upregulated in liver after nuciferine treatment (fold to experimental model group). Control, experimental model, and nuciferine-treated (n = 6 per group) groups. Data are presented as mean ± SD. ^#^*p* < 0.05 as compared to the control group; ^##^*p* < 0.01 as compared to the control group; *p < 0.05 as compared to the experimental model group; **p < 0.01 as compared to the experimental model group.

The significantly changed metabolic pathways in the NAFLD model rats were analyzed using MetaboAnalyst. Pathways with a *p-*value of less than 0.05 and an impact value of more than 0.10 were considered to be the significant pathways. Fifteen metabolic pathways were identified to be related to NAFLD. Among these pathways, linoleic acid metabolism with an impact value of 1.00 and *p-*value of 0.038, alpha-linolenic acid metabolism with impact value of 0.33 and *p-*value of 0.006, glycerophospholipid metabolism with impact value of 0.24 and *p-*value of 0.018 and arginine and proline metabolism with impact value of 0.13 and *p-*value of 0.048 were selected as the significant pathways (**Figure 6**). The summary schematic for the significant pathways was displayed in **Figure 7**. Moreover, the gene expression of the key enzymes related to the significant pathways was also investigated. The expression of *PLA2* (*p* < 0.05), *PTDSS1* (*p* < 0.01), *PTDSS2* (*p* < 0.01), *FADS2* (*p* < 0.01), *CYP2C* (*p* < 0.01), *CYP2E1* (*p* < 0.01), and *CYP3A4* (*p* < 0.01) was higher and the expression of *PEMT* (*p* < 0.05), *LCAT* (*p* < 0.01), and *ARG2* (*p* < 0.01) was lower in the experimental model group as compared to the control group (**Figure 8**). On the other hand, nuciferine treatment downregulated the expression of *PLA2* (*p* < 0.05), *PTDSS2* (*p* < 0.05), *FADS2* (*p* < 0.01), *CYP2E1* (*p* < 0.05), and *CYP3A4* (*p* < 0.01), while it upregulated the expression of *PEMT* (*p* < 0.05) and *LCAT* (*p* < 0.01) as compared to the experimental model group (**Figure 8**).

## Discussion

In this study, we established a NAFLD rat model using HFD. Consistent with previous studies (Jiang et al., 2016), our results showed that the body weight, lipid contents, and liver enzymes in the serum of animals were increased in rats receiving HFD. Moreover, rats in the experimental model group exhibited enlarged hepatocytes with severe microvesicular steatosis. Nuciferine showed a remarkable effect on the NAFLD, reflecting an improvement in hyperlipidemia and liver steatosis in the NAFLD model rats.

According to the two-hit hypothesis presented by Christopher Day and Oliver James, the hepatocytes receive first hits when excessive lipid content is accumulated in the liver with a consequent dysfunction of lipid metabolism. Moreover, dysfunction of the lipid metabolism could trigger oxidative stress and cellular damage in the hepatocytes (Chen et al., 2008). The results of the current study showed that the activities of SOD and GSH-Px were increased and the level of MDA in the liver was decreased following nuciferine treatment. MDA was the end product of lipid peroxidation and excessive O_2_ induced lipid peroxidation and caused a second hit to the hepatocytes (Vander Heiden et al., 1997). SOD and GSH-Px are antioxidant enzymes that could protect the hepatocytes from O_2_^-^. SOD catalyzed the transformation of O_2_^−^ into hydrogen peroxide (H_2_O_2_) (El-Din et al., 2014). H_2_O_2_ was then transformed into H_2_O and O_2_ following the catalysis activity of GSH-Px (El-Din et al., 2014).

Moreover, nuciferine attenuated inflammation in the NAFLD rat model, which was reflected by the downregulation of *IL-6*, *IL-1β*, and *TNF-α* mRNA expression in the liver and lower levels of IL-6, IL-1β, and TNF-α in the serum. NAFLD was accompanied by a chronic inflammatory response in the liver (Xie et al., 2019). Excessive lipid content induced the production of pro-inflammatory cytokines, including IL-6, IL-1β, and TNF-α, in the hepatocytes, which contributed to NAFLD. Thus, inhibiting the inflammatory response could alleviate NAFLD (Ding et al., 2015).

Metabolomic analysis showed that the glycerophospholipid, linoleic acid, alpha-linolenic acid, as well as arginine and proline metabolism pathways were selected as significant affected pathways in the NAFLD rat model based on their higher impact value. As an important part of biological membranes, the glycerophospholipid metabolism was involved in many biological processes such as membrane fusion, pinocytosis, and membrane transport. Therefore, dysfunction of the glycerophospholipid metabolism could negatively affect the energy metabolism in the liver (Mesens et al., 2012). Our results showed that the levels of PC and 1-acylglycerophosphocholine were decreased, whereas PE and glycerylphosphorylethanolamine were increased in the NAFLD rat model. Nuciferine treatment increased the levels of PC and 1-acylglycerophosphocholine in the serum. PCs account for 40–50% of the total phospholipids; therefore, they are the most abundant glycerophospholipids in mammalian cells. PEs account for 40% of the total phospholipids and were primarily found in the mitochondrial membrane (Van Der Veen et al., 2017). The ratio of PC to PE was decreased in the NAFLD-affected patients (Li et al., 2006). Further studies have shown that PC was significantly decreased and PE was increased in HFD-induced NAFLD models. The decreased PC to PE ratio impaired the cell membrane component and induced the permeability of the hepatocytes, which accelerated liver injury (Calzada et al., 2016). Although nuciferine treatment did not affect the PE levels in the NAFLD models, nuciferine could induce the PC to PE ratio to improve the lipid metabolism in NAFLD. Studies have demonstrated the beneficial effects of PC on hepatic inflammation. In LPS induced inflammation rat models, the combination treatment of PC and hydrocortisone could decrease the pro-inflammatory cytokine levels in serum and alleviate the infiltration of inflammatory cells in the liver (Boza et al., 2005). Similarly, our results showed that nuciferine could ameliorate the inflammatory response in a NAFLD rat model. Furthermore, the expression of enzymes (*PEMT*, *LCAT*, *PTDSS1*, *PTDSS2*, and *PLA2*) involved in the glycerophospholipid metabolism of the liver was changed in NAFLD model rats and nuciferine treatment regulated the expression of *PEMT*, *LCAT*, *PTDSS2* and *PLA2*. PC could be synthesized from choline or be transformed from PE under the catalysis of phosphatidylethanolamine N-methyltransferase (PEMT). NAFLD patients exhibited a lack of function of PEMT and the dysfunction in PEMT inhibited the transformation of PE to PC and caused the accumulation of PE (Araujo et al., 1998; Castro-Martinez et al., 2012). Lecithin–cholesterol acyltransferase (LCAT) participated in the transformation of PC to HDL-C. The expression of LCAT was decreased in the HFD-induced hyperlipidemia model (Zeng et al., 2016). Phospholipase A2 (PLA2) catalyzed the hydrolysis of PC to generate lysophosphatidylcholine (LPC). Excessive LPC induced apoptosis of the hepatocytes (Han et al., 2008). PE and PC were transformed into phosphatidylserine under the catalysis of phosphatidylserine synthase (PTDSS). Microarray analysis revealed that the expression of PTDSS was increased in diabetic model rats. Downregulation of PTDSS induced the levels of PC (Choi et al., 2012).

Linoleic acid and alpha-linolenic acid metabolisms are associated with metabolic syndrome. The levels of linoleic acid, dihomo-gamma-linolenate, rumenic acid and alpha-linolenic acid were decreased in the NAFLD rat model, whereas these metabolites exhibited opposite trends following nuciferine treatment. Linoleic acid, dihomo-gamma-linolenate, and alpha-linolenic acid are unsaturated fatty acids (UFAs) and have been demonstrated to have beneficial effects on metabolic disorders. Linoleic acid has been demonstrated a lipid-lowering effect through inducing the transformation of TC (Koo, 2013). Treatment of conjugated linoleic acid ameliorated obesity through reducing lipogenesis and increasing lipolysis and fat oxidation (Wang et al., 2017). Dietary oils rich in linoleic acid and alpha-linolenic acid improved the obesity-related glomerulopathy through reducing the visceral adiposity and glomerular damage (Caligiuri et al., 2014). The current results also showed that the gene expression related to linoleic acid (*CYP2C*, *CYP2E1*, and *CYP3A4*) and alpha-linolenic acid (*FADS2*) metabolism was changed in the NAFLD model group, whereas the expression of *CYP2E1*, *CYP3A4*, and *FADS2* exhibited opposite trends following nuciferine treatment. Cytochrome P-450 (CYP) enzyme system, which was primarily expressed in the liver, involved several metabolic processes of endogenous and exogenous compounds and was demonstrated to be closely related to the progression of NAFLD (Bell et al., 2010). CYP2C, CYP2E1, and CYP3A4 catalyzed the oxidation of linoleic acid, generating 12(13)-EpOME and 9(10)-EpOME. The expression of CYP2C (Fisher et al., 2009), CYP2E1 (Bell et al., 2011) and CYP3A4 (Fisher et al., 2009) was upregulated in the liver of the NAFLD model. Increased activities of the CYP enzymes induced the peroxidation of the lipid contents and increased the levels of MDA in the hepatocytes (Bell et al., 2010). The anti-oxidative mechanisms of nuciferine in NAFLD might be related to inhibiting the expression of the CYP enzymes in the liver. Alpha-linolenic acid could be transformed into stearidonic acid under the catalysis of FADS2. The expression of FADS2 was upregulated in obese patients. FADS2 induced the inflammatory response in the adipose tissue, which also supported the anti-inflammatory properties of nuciferine in NAFLD (Vaittinen et al., 2015).

The metabolism of amino acids was an important component of NAFLD-related metabolic disorders. Levels of L-arginine and L-proline were increased in the NAFLD rat model, while nuciferine treatment decreased the L-proline levels. L-arginine transformed into L-ornithine and urea *via* the urea cycle to decrease ammonia. L-ornithine could transform into L-proline and, in turn, L-proline could transform into pyruvate to enter the citrate cycle. The dysfunction of arginine and proline metabolism has been observed in a NAFLD model (Sun et al., 2020). The current study also showed that the expression of ARG2 was upregulated in NAFLD model rats. ARG2 is a key enzyme in the urea cycle and catalyzes the transformation of L-arginine into L-ornithine. Dysfunction in the urea cycle induced the accumulation of L-arginine and caused hyperammonemia. High levels of blood ammonia could activate hepatic stellate cells to induce hepatic fibrosis and could contribute to NAFLD progression (Thomsen et al., 2018).

In conclusion, our study demonstrated the therapeutic effects of nuciferine in a NAFLD rat model. Untargeted metabolomic analysis indicated significant dysfunction in the glycerophospholipid, linoleic acid, alpha-linolenic acid, arginine, and proline metabolism pathways. The mechanism of nuciferine in NAFLD might be related to the improvement of these dysregulated metabolic pathways.

## Data Availability Statement

The raw data supporting the conclusions of this article will be made available by the authors, without undue reservation.

## Ethics Statement

The animal study was reviewed and approved by Animal Ethics Committee in Tianjin University of TCM (SYXK2019-0006).

## Author Contributions

HC wrote the manuscript. HC, YL, MC, JL, and HF conducted animal experiments. YL, RS, and HC finished molecular bioassays. WW, JM, XL, HW, and ZZ provided technical guidance for the whole work. All authors contributed to the article and approved the submitted version.

## Funding

This work was supported by National Science Foundation of China (81560772), Natural Science Foundation of Tianjin (17JCYBJC42800), Science and Technology Projects in Key Fields of Traditional Chinese Medicine of Tianjin Municipal Health Commission (No.2020006), and Public Welfare Research Projects in Jiaxing (SQ2018001355).

## Conflict of Interest

The authors declare that the research was conducted in the absence of any commercial or financial relationships that could be construed as a potential conflict of interest.
