# Supplementary material for: Untargeted Metabolomic Analysis of the Effects and Mechanism of Nuciferine Treatment on Rats With Nonalcoholic Fatty Liver Disease
Source: Front Pharmacol. 2020 Jun 9;11:858. doi: 10.3389/fphar.2020.00858 (PMC7295953; doi:10.3389/fphar.2020.00858)
Supplement: Supplementary file 1 [file DataSheet_1.docx]

**a**


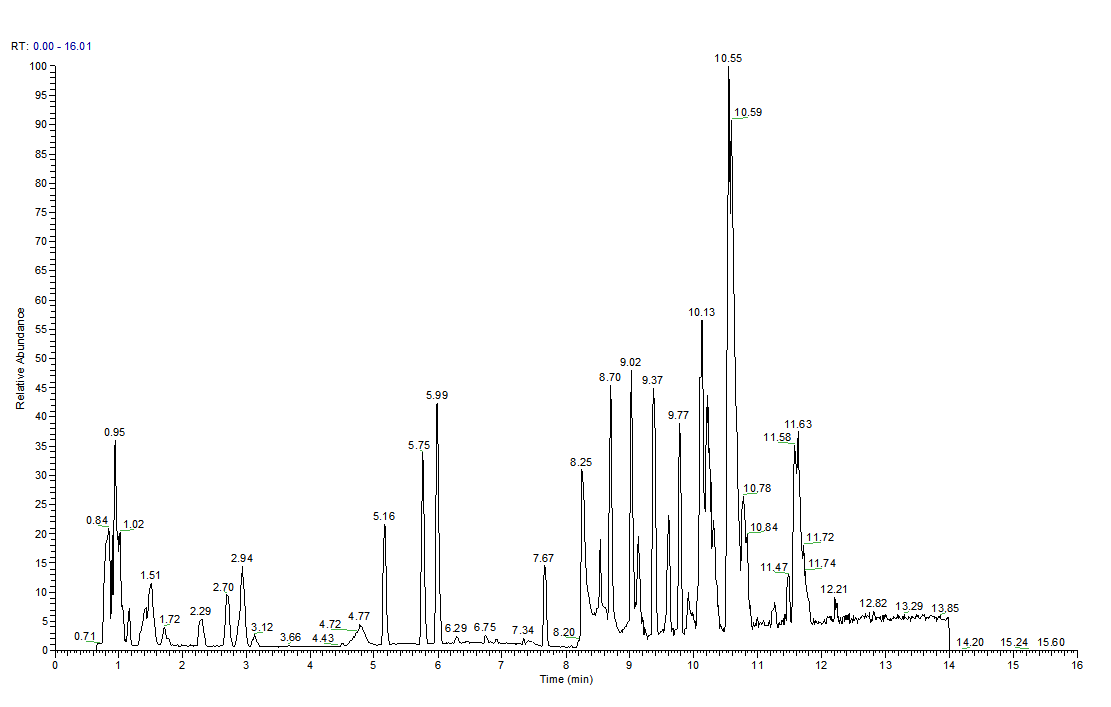


**b**


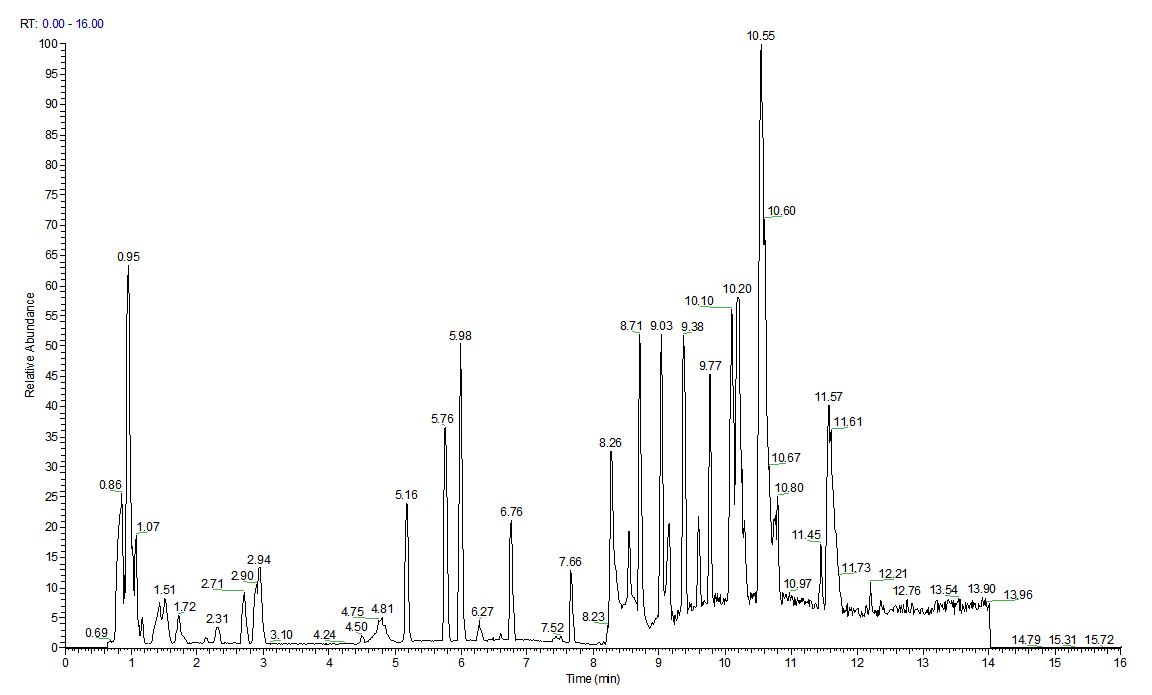


**c**

**
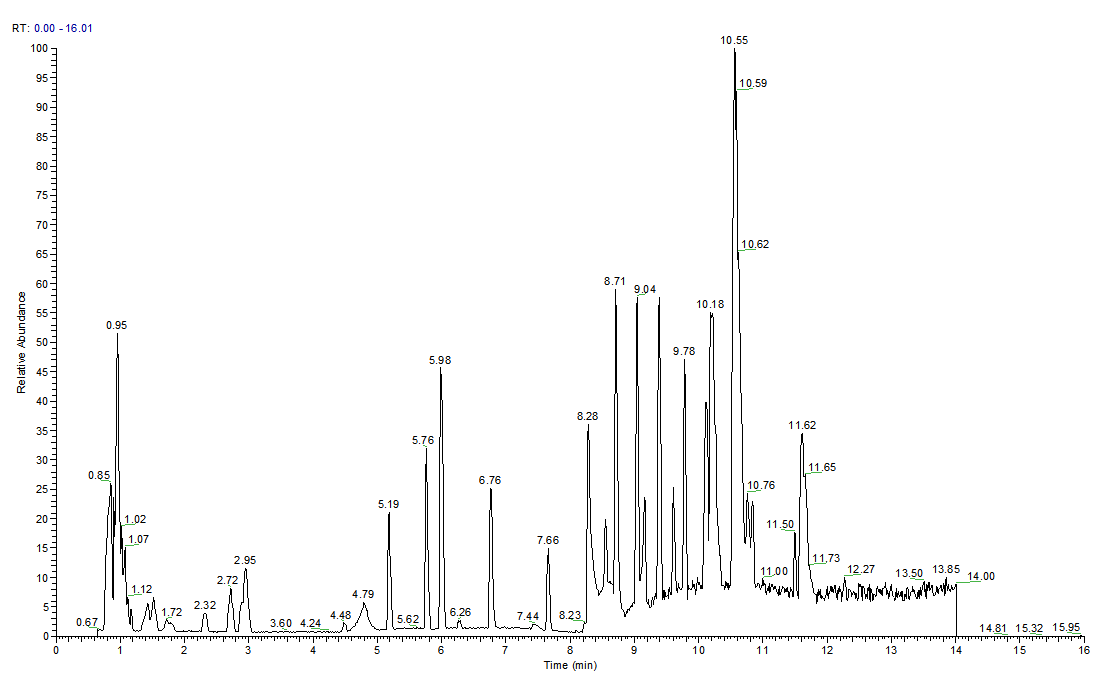
**

**sFigure 1:** The typical based peak intensity (BPI) chromatograms of serum samples obtained in positive ion mode. (a) Control group; (b) Model group; (c) Nuciferine group.

**a**

**
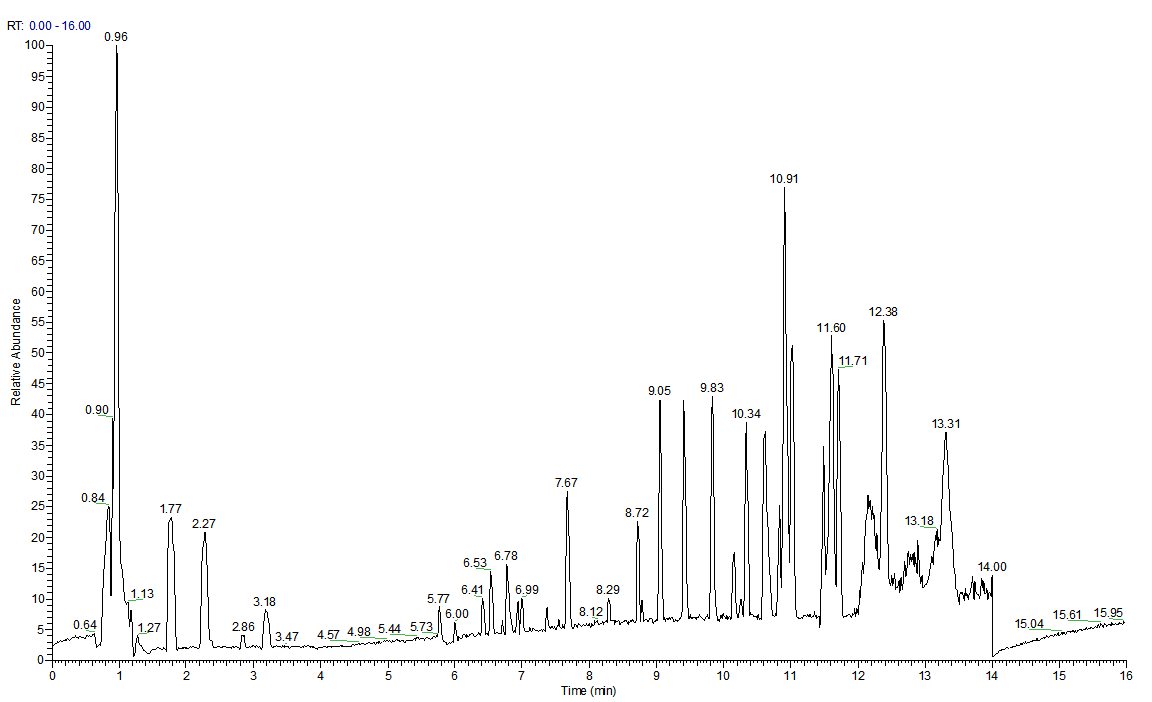
**

**b**


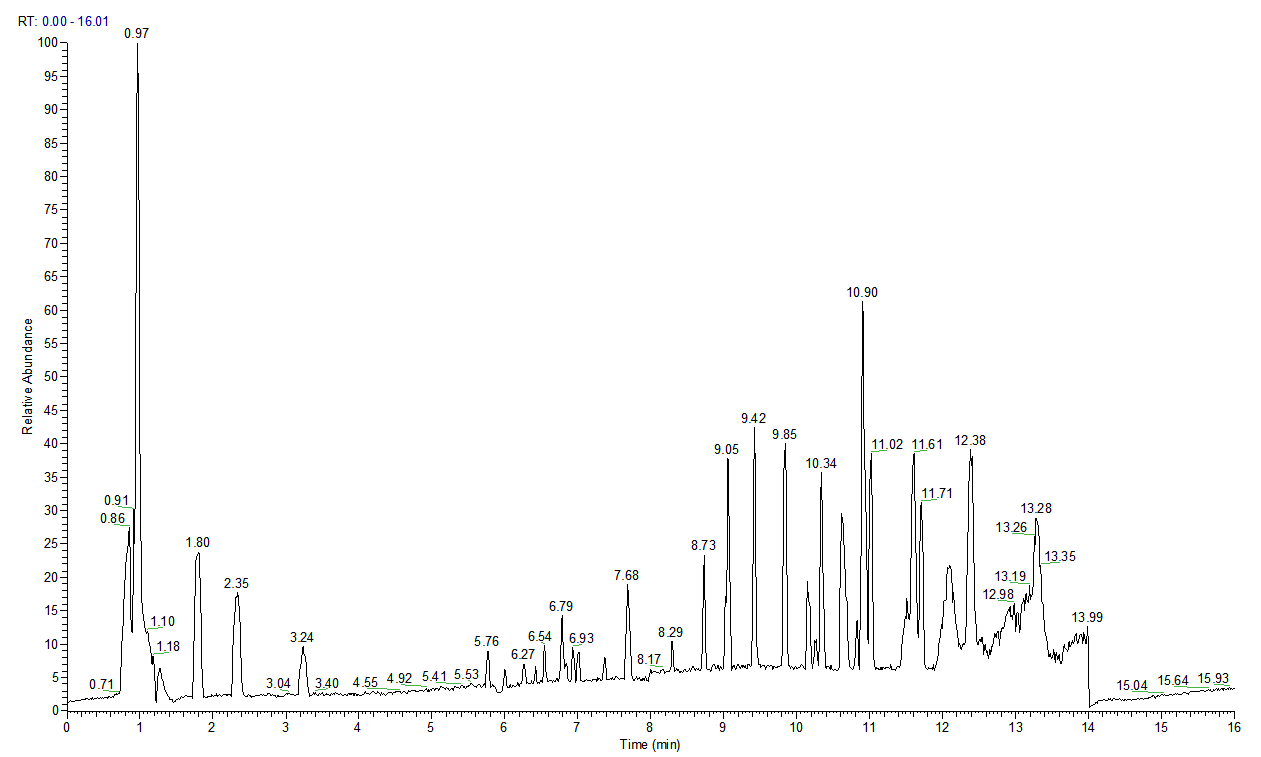


**c**

**
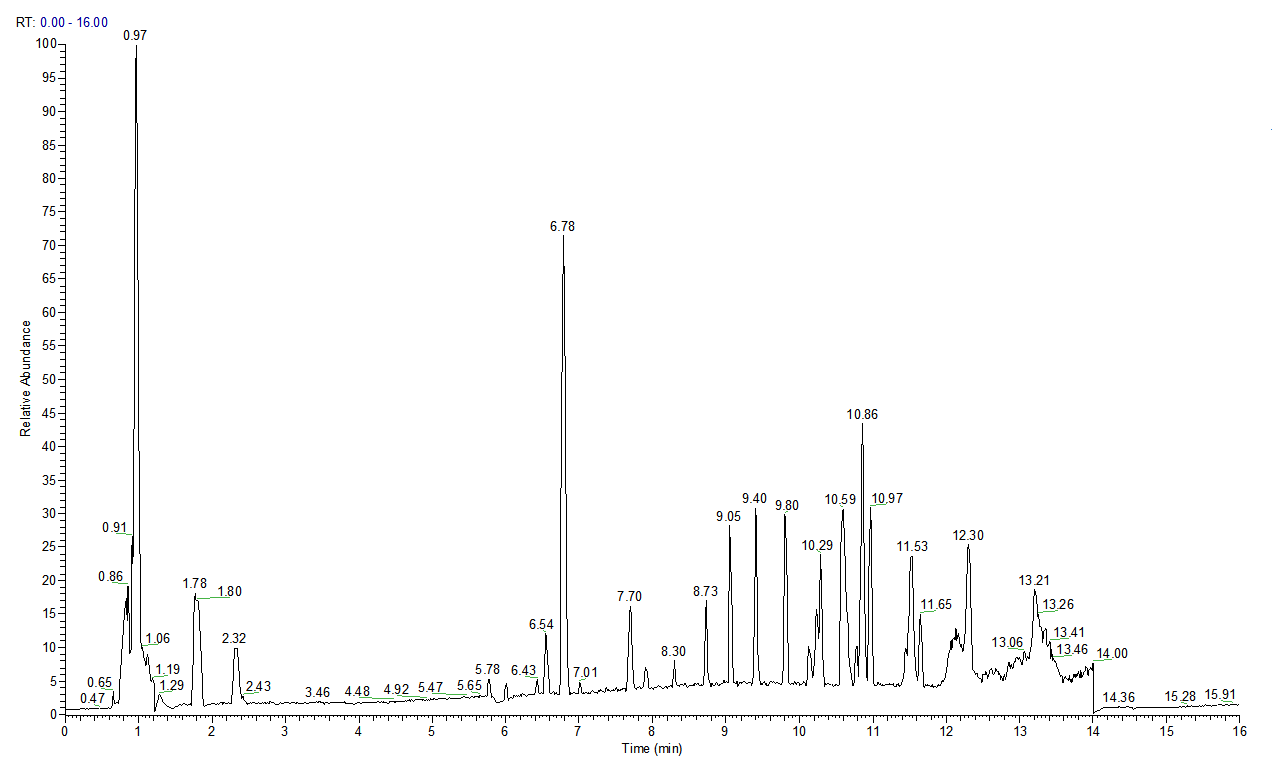
**

**sFigure 2:** The typical based peak intensity (BPI) chromatograms of serum samples obtained in negative ion mode. (a) Control group; (b) Model group; (c) Nuciferine group.
